# Sex differences in the response to resistance exercise training in older people

**DOI:** 10.14814/phy2.12834

**Published:** 2016-06-28

**Authors:** Mariasole Da Boit, Rachael Sibson, Judith R. Meakin, Richard M. Aspden, Frank Thies, Arduino A. Mangoni, Stuart Robert Gray

**Affiliations:** ^1^ Department of Life SciencesUniversity of DerbyDerbyUnited Kingdom; ^2^ Institute of Medical SciencesUniversity of AberdeenAberdeenUnited Kingdom; ^3^ Exeter MR Research CentreUniversity of ExeterExeterUnited Kingdom; ^4^ Department of Clinical PharmacologySchool of MedicineFlinders UniversityAdelaideAustralia; ^5^ Institute of Cardiovascular and Medical SciencesUniversity of GlasgowGlasgowUnited Kingdom; ^6^ Present address: Institute of Medical SciencesUniversity of AberdeenAberdeenUnited Kingdom

**Keywords:** Aging, exercise, muscle adaptation, sexual dimorphism

## Abstract

Resistance exercise training is known to be effective in increasing muscle mass in older people. Acute measurement of protein metabolism data has indicated that the magnitude of response may differ between sexes. We compared adaptive responses in muscle mass and function to 18 weeks resistance exercise training in a cohort of older (>65 years) men and women. Resistance exercise training improved knee extensor maximal torque, 4 m walk time, time to complete five chair rises, muscle anatomical cross‐sectional area (ACSA) and muscle quality with no effect on muscle fat/water ratio or plasma glucose, insulin, triacylglycerol, IL‐6, and TNF‐*α*. Differences between sexes were observed for knee extensor maximal torque and muscle quality with greater increases observed in men versus women (*P* < 0.05). Maximal torque increased by 15.8 ± 10.6% in women and 41.7 ± 25.5% in men, whereas muscle quality increased by 8.8 ± 17.5% in women and by 33.7 ± 25.6% in men. In conclusion, this study has demonstrated a difference in the magnitude of adaptation, of some of the outcome measures employed, in response to 18 weeks of resistance exercise training between men and women. The mechanisms underlying this observation remain to be established.

## Introduction

The loss of skeletal muscle mass and function can occur in many conditions, such as sarcopenia (age related atrophy), cachexia (wasting associated with disease), and general disuse (Baumgartner et al. 1998; Cruz‐Jentoft et al. 2010). Focusing on aging, “healthy aging” is associated with a progressive loss of skeletal muscle mass, approximately 0.5–2% per annum, and skeletal muscle strength, around 3% per annum, after reaching 40–50 years of age (Baumgartner et al. 1998; Clark and Manini 2008). The incidence of sarcopenia has been found to be between 4.6 and 7.9% in community dwelling older men and women in the UK (Patel et al. 2013). In the year 2000, the healthcare costs associated with sarcopenia were estimated to $18.5 billion in the USA (Janssen et al. 2004), with this figure likely to be higher currently due to the increasing age of the population. The major consequences of sarcopenia include the concomitant loss of functional abilities, such as the ability to rise from a chair or step on to a bus, an increase in the likelihood of falls and subsequent hospitalization, loss of independence, and a decrease in the quality of life (O'Loughlin et al. 1993).

Currently, there are no therapeutic interventions that can prevent this loss of muscle mass and function which occurs with aging. However, resistance exercise can be effective in older people, even nonagenarians (Fiatarone et al. 1990), although to a lesser extent than in younger people due to the so called anabolic resistance (Wackerhage and Rennie 2006). Another factor which may determine the magnitude of response to anabolic stimuli, such as resistance exercise and nutrition, is sex although this is rarely accounted for in such studies. The first study to investigate this found that in response to a bout of resistance exercise muscle protein synthesis (MPS), measured for 2 h after exercise, was the same regardless of sex in healthy older people (Dreyer et al. 2010). In another study in obese older men and women it was demonstrated that prior to any training basal MPS was higher in women compared to men but while the consumption of a mixed‐meal increased MPS in men, it had no effect in women. After 3 months of resistance exercise training it was shown that in both men and women basal MPS was increased, with no effect on post mixed‐meal MPS, but the increase was larger in men (Smith et al. 2012b). However, we know that acute postexercise measures of MPS do not reflect long‐term muscle hypertrophy (Mitchell et al. 2014), highlighting the need for longer term studies. Longer term studies are lacking and the current data ambiguous. In a small study (9 men and 5 women) relative increases in muscle fiber hypertrophy and maximal strength, in response to 26 weeks of resistance exercise training, were greater in older men compared to older women (Bamman et al. 2003). In a larger study (29 men and 24 women) it was shown that, in response to 24 weeks of resistance exercise training, relative increases in muscle mass and function were similar between men and women (Leenders et al. 2013b). Interpretation of these results are, however, confounded as the participants had been randomly assigned to either 15 g/day protein or placebo (Leenders et al. 2013a) and, although in that study there was no effect of protein on muscle strength/function overall, we cannot be sure these findings reflect purely the effects of exercise alone (i.e., 2 variables were different between participants: sex and supplement).

The aim of this study, therefore, was to determine whether skeletal muscle adaptations to a long‐term resistance exercise training differ between older men and women. Due to the aforementioned studies, we hypothesize that resistance exercise training will result in a greater anabolic response in older men compared to older women.

## Methods

### Participants

Ten women and 13 men (aged 71.3 ± 4.1 years, height 166.6 ± 9.4 cm, weight 70.1 ± 12.3 kg; mean ± SD) volunteered to participate in the study. Participants were recruited via advertisements placed in local newspapers and magazines. Participants were classified as medically stable (Greig et al. 1994), that is, free from cardiac illness, cancer, arthritis, respiratory disease, metabolic disease, recent fractures, and loss of mobility. Furthermore, participants were not on daily analgesia and were not consuming any nutritional supplements or participating in regular exercise. Two participants were taking medication: one female volunteer (ACE inhibitors) for mild hypertension, and one male volunteer (allopurinol) for gout. These participants were recruited as part of a randomized controlled trial (UKCRN ID 13024) investigating the effects of fish oil consumption on the adaptive response to resistance exercise training and were the control group within this trial. As part of this trial, participants from the control group consumed 3.0 g of safflower oil per day for 18 weeks, which was neutral with regards to red blood cell fatty acid composition (Table 1). The study was approved by the University of Aberdeen College of Life Sciences and Medicine Ethics Review Board (CERB/2011/6/644). Participants were made aware of the aims, risk, and potential discomfort associated with the study prior to providing written informed consent.

**Table 1:** Red blood cell fatty acid composition in men and women before and after the 18‐week resistance exercise period

Fatty acid (% total fatty acids)	Men (*n* = 13)		Women (*n* = 10)	
	Baseline	18 weeks	Baseline	18 weeks
Palmitic (C16:0)	23.56 ± 1.01	23.58 ± 1.11	24.17 ± 0.80	24.01 ± 1.91
Palmitoleic (C16:1)	0.69 ± 0.47	0.67 ± 0.51	0.80 ± 0.61	0.44 ± 0.43
Stearic (C18:0)	14.22 ± 0.70	14.16 ± 1.15	14.11 ± 1.28	14.79 ± 1.09
Oleic (C18:1)	17.33 ± 0.99	17.34 ± 1.14	18.50 ± 2.57	18.09 ± 2.86
Linoleic (C18:2)	15.58 ± 2.53	16.73 ± 2.62	15.46 ± 2.27	16.62 ± 3.09
Eicosatrienoic (C20:3)	2.41 ± 0.97	2.03 ± 0.89	1.80 ± 0.60	1.55 ± 0.46
Arachidonic (C20:4)	12.99 ± 2.04	12.89 ± 2.25	12.87 ± 1.29	12.53 ± 2.39
Eicosapentaenoic (C20:5)	1.44 ± 0.97	1.50 ± 1.33	1.47 ± 0.71	1.74 ± 0.50
Lignoceric (C24:0)	1.83 ± 0.26	1.56 ± 0.57	1.53 ± 0.23	1.61 ± 0.50
Nervonic (C24:1)	2.12 ± 0.35	1.84 ± 0.66	1.79 ± 0.26	1.87 ± 0.46
Docosapentaenoic (C22:5)	2.67 ± 0.27	2.44 ± 0.88	2.32 ± 0.43	2.93 ± 1.00
Docosahexaenoic (C22:3)	5.16 ± 1.19	5.26 ± 1.40	5.18 ± 0.81	5.82 ± 0.62

### Study protocol

Upon entry to the study baseline measurements of body weight, height, and physical activity levels (IPAQ–short form) were performed, knee extensor isometric strength was measured, the short performance physical battery test (SPPB) was administered, quadriceps muscle volume and fat content were measured by magnetic resonance imaging (MRI), and a fasting blood sample was collected. Then participants undertook a supervised resistance exercise training program of the lower limbs for 18 weeks, with two sessions per week. All participants completed all 36 sessions. As seven male participants went on holiday for an average of 1 week, their training periods were extended by the same period. Each session consisted of participants performing leg extension, leg press, leg curl, and calf raise exercise (4 sets of 9 repetitions at 70% of their one‐repetition maximum [1RM]). The 1RM was retested every 6 weeks and the load readjusted accordingly. Repeated measurements of body weight, knee extensor isometric strength, and the SPPB were made every 6 weeks, whereas blood samples and MRI measures were repeated at 18 weeks.

### Measurements

#### Knee extensor isometric strength

Knee extensor isometric strength of the right leg was determined during a maximal voluntary contraction (MVC) with the participant seated on a Biodex dynamometer at a knee angle of 73 degrees. Subjects were secured on the seat using seatbelts and the settings recorded and reproduced during successive measurements. Each MVC was repeated a minimum of three times and the highest values used for subsequent analysis.

#### Short performance physical battery test

The SPPB consists of tests of balance, walk speed, and timed chair standing (Guralnik et al. 2000). The balance tests required participants to maintain a side‐by‐side, semitandem, and tandem stance for 10 sec. All participants were able to complete these three tests for the full 10 sec and so these data are not presented. The chair standing test involved participants rising from a chair with their arms across their chest five consecutive times, and this was repeated three times with the time recorded. From a standing start participants were instructed to complete three separate 4 m walks at the fastest pace possible and the time recorded. For both tests the fastest of the three attempts was used for subsequent analysis.

#### Magnetic resonance imaging

All scans were carried out on a Philips Achieva 3.0 Tesla whole‐body MRI scanner using a 16‐channel SENSE XL Torso coil. Participants lay in a supine position with their feet going into the bore of the scanner first, with the knee extended but in a relaxed state. Velcro straps were used to keep the feet and legs close together with the ankle angle for both feet neutral. The acetabulum and knee joint line were used as primary reference landmarks to identify positions of the scans. A cod liver oil capsule was attached with surgical tape to the midpoint of the thigh to provide a point of reference for scans, particularly when the participants were too tall for the full thigh to be scanned in a single sequence. High‐resolution T_1_‐weighted turbo spin echo images were collected contiguously over the length of the thigh (Repetition time 400 msec, echo time 15 msec, slice thickness 10 mm and flip angle 90 degrees) for the calculation of quadriceps muscle anatomical cross‐sectional area (ACSA). A dual‐echo scan giving both in‐phase and out‐phase T1‐weighted images in a single acquisition (Repetition time 190 msec, echo time 2.3/3.5 msec, slice thickness 10 mm and flip angle 70 degrees) was used for the determination of muscle fat/water ratio. Specifically, the in‐phase image (sum of fat and water) and the out‐phase image (difference of fat and water) were combined to obtain a water‐only and a fat‐only images. These two images were then used to extrapolate direct image‐based water and fat quantitation, via average signal intensity. MR images were analyzed using ImageJ (version 1.49, Bethesda, MD, NIH). We analyzed 5 slices (the midpoint slice and 2 immediately inferior and superior to this) with ACSA determined by manually drawing round the quadriceps muscle with fat/water ratio also determined in this manually drawn region of interest. We chose not to analyze all slices as it became problematic to accurately draw around the muscles near to the inferior and superior regions of the thigh and we found five slices to give us the most reproducible measure of ACSA. Muscle quality was calculated as force (knee extensor isometric strength) per unit ACSA.

#### Blood sampling

Blood samples were collected from a vein in the antecubital fossa into K+EDTA vacutainers, placed on ice and processed within 30 mins. Samples were centrifuged for 10 min at 4°C at 2000*g* and the erythrocyte and plasma aliquoted and stored at −80°C until analysis. Plasma glucose and triacylglycerol were measured in duplicate using a commercially available spectrophotometric assay following the manufacturer's instructions (Randox). Insulin, interleukin‐6 (IL‐6), and tumor necrosis factor‐alpha (TNF‐*α*) were measured using commercially available ELISA assays according to the manufacturer's instructions (Insulin – Mercodia and IL‐6 and TNF‐*α* – RnD systems). Lipids were extracted from red blood cells with chloroform:methanol (2:1, by vol) with butylated hydroxytoluene present (0.01%) to prevent oxidation of fatty acids. Fatty acid methyl esters were prepared by incubation with 14% boron trifluoride in methanol at 80°C for 60 min. Fatty acid methyl esters were separated and identified by gas chromatography by comparison with standards run previously.

### Statistical analysis

Data analysis was carried out using SPSS software (v19, IBM Business Analytics; IBM, Hampshire, UK). Data were analyzed using a two‐way (time and sex) repeated measures ANCOVA, with baseline values as the covariate. Percentage changes (from 0 to 18 weeks) were calculated and compared between sexes using independent *t*‐test. Statistical significance was accepted as *P *<* *0.05. Data are expressed as mean ± SD.

## Results

### Baseline characteristics

As shown in Table 2, there were no significant differences between men and women in age, weight or body mass index (BMI) at the beginning of the study. Men were taller (*P *<* *0.05) than women, as expected. The resistance exercise program did not alter weight or BMI. Physical activity levels, according to the IPAQ classification, were as follows: 11 participants in category 1 (inactive): five women and six men; 10 subjects in category 2 (minimally active): four women and six men; and two subjects in category 3 (health enhancing physical activity) one woman and one man.

**Table 2:** Baseline characteristics of men and women

	Men (*n* = 13)	Women (*n* = 10)
Age (years)	71.5 ± 5.1	70.9 ± 2.6
Height (cm)	171.6 ± 8.0	160.0 ± 6.7^a^
Weight (kg)	73.2 ± 11.5	66.0 ± 12.7
BMI (kg/m^2^)	24.7 ± 2.6	25.8 ± 4.6

a Denotes a significant (*P* < 0.05) difference between men and women. BMI, body mass index.

### Muscle strength and functional abilities

**Figure 1:**
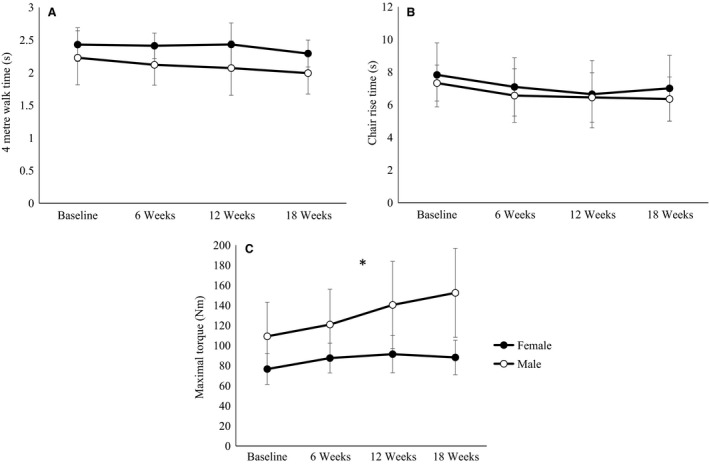
Four‐meter walk time (A), chair rise time (B), and knee extensor maximal torque (C) in men and women before and after the 18‐week resistance exercise period. * denotes a significant (*P* < 0.05) interaction effect.

The resistance exercise program resulted in a significant increase in maximal torque (*P *<* *0.05) and faster times to complete the 4 m walk and the five chair rises (*P *<* *0.05) in both sexes. An interaction effect with sex and time was observed for maximal torque (*P *<* *0.05) but not for walk or chair rise time. From baseline to 18 weeks maximal torque increased by 15.8 ± 10.6% in women, whereas there was a greater increase (*P *<* *0.05) of 41.7 ± 25.5% in men. The time to complete the 4 m walk improved by 6.5 ± 8.6% in women and by 10.0 ± 9.3% in men, with no differences in these improvements between sexes (*P *>* *0.05). We observed a decrease in the time required to complete five chair rises of 13.9 ± 15.3% in women and 13.6 ± 12.1% in men, with no differences between sexes (P > 0.05) (Fig. 1).

### Muscle cross‐sectional area, fat/water ratio, and quality

The 18 weeks of resistance exercise training resulted in significant increases in muscle ACSA and muscle quality–force per unit ACSA (both *P *<* *0.05), with no effect on muscle fat/water ratio (*P > *0.05) (Table 3). There were no interaction effects with sex for muscle ACSA or muscle fat, but an interaction effect was observed for muscle quality (*P *<* *0.05). Muscle ACSA increased by 2.0 ± 3.6% in women and by 3.6 ± 2.9% in men, with no difference between sexes (*P* > 0.05). Muscle fat/water ratio tended to increase by 1.2 ± 3.6% in women and decreased by 2.7 ± 4.7% in men (*P* = 0.09). Muscle quality increased by 8.8 ± 17.5% in women with a greater (*P *<* *0.05) increase of 33.7 ± 25.6% in men.

**Table 3:** Muscle CSA, fat, and quality in men and women before and after the 18‐week resistance exercise period

	Men (*n* = 12)		Women (*n* = 10)	
	Baseline	18 weeks	Baseline	18 weeks
ACSA (cm^2^)	56.7 ± 8.7	58.8 ± 8.6	37.1 ± 5.7	37.8 ± 6.2
Muscle fat (%)	8.2 ± 2.0	8.0 ± 1.9	8.8 ± 2.1	8.9 ± 2.1
Muscle quality (torque per cm^2^)	1.93 ± 0.49	2.58 ± 0.58^a^	2.11 ± 0.55	2.22 ± 0.42^a^

a Denotes a significant (*P* < 0.05) interaction effect.

### Plasma measures

Plasma glucose, insulin, triacylglycerol, IL‐6, TNF‐*α,* and calculated HOMA‐ IR remained unchanged after intervention (Table 4).

**Table 4:** Plasma measures of glucose, insulin, triacylglycerol, IL‐6, TNF‐*α,* and calculated HOMA‐IR in men and women before and after the 18‐week resistance exercise period

	Men (*n* = 11)		Women (*n* = 9)	
	Baseline	18 weeks	Baseline	18 weeks
Glucose (mmol/L)	5.33 ± 0.49	5.14 ± 0.35	6.07 ± 1.44	6.21 ± 1.64
Insulin (mU/L)	6.11 ± 2.66	5.71 ± 2.52	6.96 ± 4.67	6.02 ± 3.18
HOMA‐IR	1.43 ± 0.61	1.31 ± 0.61	1.89 ± 1.31	1.68 ± 0.95
Triacylglycerol (mmol/L)	0.86 ± 0.25	0.89 ± 0.31	1.29 ± 0.87	1.03 ± 0.32
IL‐6 (pg/mL)	1.34 ± 0.66	0.88 ± 0.24	1.77 ± 1.11	1.96 ± 1.02
TNF‐*α* (pg/mL)	4.91 ± 2.61	4.62 ± 2.05	4.81 ± 2.13	4.67 ± 1.89

## Discussion

This study has demonstrated for that some of the adaptations that occur with 18‐week resistance exercise training differ in magnitude between sexes. Specifically, the increase in knee extensor muscle strength and muscle quality was greater in men compared to women. Improvements in functional abilities (walk and chair rise speed) and muscle mass were not different between the sexes. The study, however, was not powered to detect differences in these variables and so further work is needed in this area. These findings suggest that older women may require a greater resistance exercise stimulus, if possible, to achieve the same improvements as seen in men. The mechanisms underlying these observations are not known but merit further consideration.

With the major role of muscle mass and function in healthy aging it is therefore important to understand factors which play a role in determining muscle mass and function and in this regard sex is of clear importance. Lindle et al. (1997) investigated the effect of age and sex on muscle strength. This research demonstrated that while men have greater muscle strength when younger, after reaching around 40 years of age muscle strength decreases in both sexes; the percentage rate of decline was similar in both men and women. However, as women begin at a lower initial muscle strength they cross the “disability threshold”, where functional impairments become evident, earlier and thus, although women live longer than men, they spend more time in a disabled state (Dunlop et al. 1997). The findings of this study, larger increase in muscle strength in men, are in agreement with some (Bamman et al. 2003) but not all of the previous work in this area (Leenders et al. 2013b), and may indicate that sex‐specific resistance exercise strategies are needed. Further work is needed in this area.

This study found that, in response to resistance exercise training, not only did muscle strength not increase to the same extent in women as men but that the increase in muscle quality was also limited. Muscle quality refers to the maximal force relative to the mass of the muscle and is determined by many factors such as the composition of the muscle architecture, fat and connective tissue infiltration, and neuromuscular properties (Doherty et al. 1993; Frontera et al. 2000; Doherty 2003). Declines in muscle quality have been demonstrated to occur with age (Goodpaster et al. 2006) and to be associated with impairments in physical function (Hairi et al. 2010). Resistance exercise is known to be effective in increasing muscle quality in both young and old people and in one study it was shown that these increases in muscle quality are similar between older men and women (Ivey et al. 2000). This is in disagreement with the findings of this study likely due to the short nature of the study of Ivy and colleagues and may reflect the mechanism underlying the improvements in muscle quality. In the first few weeks of resistance exercise training much of the increase in muscle quality is likely due to neuromuscular improvements such as an increase in motor unit recruitment (Hakkinen et al. 1998). In contrast, the latter increases in muscle quality are likely due to factors such as changes in fiber type, fat and connective tissue infiltration, muscle architecture, and tendon adaptations (Aniansson and Gustafsson 1981; Degens et al. 2009; Erskine et al. 2011). It may be that while initial neuromuscular adaptations are similar between sexes, these latter adaptations do not occur to the same extent in women. This hypothesis remains to be tested.

Muscle ACSA increased in both sexes during the 18‐week resistance exercise program employed in this study, with no differences between the sexes. This is generally not in agreement with the data investigating sex differences in muscle protein metabolism in older people. While Dreyer et al. (2010) found that exercise has similar effects on MPS in young men and women, the work of Smith et al. (2012a,b) demonstrated that, while basal MPS was higher in old women versus old men, the increase in MPS in response to resistance exercise training was less in the old women. There were limitations in the study of Smith et al., which the authors acknowledged, with regards to the duration over which MPS was measured (3.5 h) and the timing of the measurement after the last exercise bout (15–21 h where the acute effect of exercise on MPS would still be present) (Rennie and Tipton 2000). This again highlights the need to rely on long‐term studies investigating muscle mass and function and not solely MPS which, although of clear physiological importance, is only one factor among many which contribute to changes in muscle mass and function. On the other hand, the primary outcome of this study was muscle knee extensor torque, and not muscle ACSA, and with the relatively large variation in response to resistance exercise and the fact that changes in ACSA are more subtle and require longer periods of time we may not have been statistically powered to detect differences in muscle ACSA between sexes (a numerically larger increase of 3.6 ± 2.9% in men compared to 2.0 ± 3.6% in women). We cannot therefore rule of more subtle differences, compared to the clear differences in torque and quality, in the hypertrophic response to resistance exercise between sexes in older people.

The mechanism(s) underlying the differences in the adaptive response to resistance exercise remain to be established. One potential area which may be worthy of further study is in relation to the concept of “muscle memory”. This refers to the observation that prior exercise training confers an ability to increase muscle strength more quickly upon an individual beginning resistance exercise training again (Staron et al. 1991; Taaffe and Marcus 1997). While the mechanisms underlying this phenomenon are not clear they are likely to involve both neuromuscular and myonuclear adaptations (Rutherford and Jones 1986; Bruusgaard et al. 2010). Unfortunately, we did not take a prior exercise history in our current participants. However, data from the Medical Research Council National Survey of Health and Development (NHSD) (1946 British Cohort) indicate that a large gap in moderate to vigorous physical activity (with men having higher levels) has been present throughout the adult life of this cohort which includes participants of a similar age to the participants in this study (Golubic et al. 2014). Therefore, it may be that a higher level of “muscle memory” in the men of this study allowed them to improve their strength and muscle quality to a greater extent than women. With this study small in participant numbers and the aforementioned issues with previous studies in this area large‐scale high‐quality studies are needed to confirm the sex differences, observed in this study, in the increases in muscle mass and function in response to resistance exercise and to evaluate the mechanisms underlying these observations.

In conclusion, this study has demonstrated that in response to 18 weeks of supervised resistance exercise training older men increase muscle strength and quality to a greater extent than women. From a practical point of view, it may be that older women require a greater resistance exercise stimulus than men, for the same adaptive response.

## Conflict of Interests

No conflicts of interest.
